# Usefulness in Developing an Optimal Training Program and Distinguishing between Performance Levels of the Athlete’s Body by Using of Thermal Imaging

**DOI:** 10.3390/ijerph17165698

**Published:** 2020-08-06

**Authors:** Teresa Kasprzyk-Kucewicz, Agnieszka Szurko, Agata Stanek, Karolina Sieroń, Tadeusz Morawiec, Armand Cholewka

**Affiliations:** 1 Faculty of Science and Technology, University of Silesia, Bankowa 12, St., 40-007 Katowice, Poland; agnieszka.szurko@us.edu.pl (A.S.); armand.cholewka@us.edu.pl (A.C.); 2 Department of Internal Medicine, Angiology and Physical Medicine, Faculty of Medical Sciences in Zabrze, Medical University of Silesia, Batorego 15 St., 41-902 Bytom, Poland; agata.stanek@gmail.com; 3 Chair of Physiotherapy, Department of Physical Medicine, Faculty of Health Sciences, Medical University of Silesia in Katowice, Medyków Street 12, 40-752 Katowice, Poland; ksieron@hot.pl; 4 Division of Medicine and Dentistry, Department of Oral Surgery, Medical University of Silesia, Pl. Akademicki 17, 41-902 Bytom, Poland; tmorawiec@sum.edu.pl

**Keywords:** thermal imaging, thermoregulation, sport, sports medicine

## Abstract

The goal of the training is to enable the body to perform prolonged physical effort without reducing its effectiveness while maintaining the body’s homeostasis. Homeostasis is the ability of the system to maintain, in dynamic balance, the stability of the internal environment. Equally as important as monitoring the body’s thermoregulation phenomena during exercise seems to be the evaluation of these mechanisms after physical effort, when the athlete’s body returns to physiological homeostasis. Restoring homeostasis is an important factor in body regeneration and has a significant impact on preventing overtraining. In this work we present a training protocol using a rowing ergometer, which was planned to be carried out in a short time and which involves working the majority of the athlete’s muscles, allowing a full assessment of the body’s thermal parameters after stopping exercise and during the body’s return to thermal equilibrium and homeostasis. The significant differences between normalized mean body surface temperature obtained for the cyclist before the training period and strength group as well as before and 10 min after training were obtained. Such observation seems to bring indirectly some information about the sportsperson’s efficiency due to differences in body temperature in the first 10 min of training when sweat does not play a main role in surface temperature. Nearly 1 °C drop of mean body temperature has been measured due to the period of training. It is concluded that thermovision not only allows you to monitor changes in body temperature due to sports activity, but also allows you to determine which of the athletes has a high level of body efficiency. The average maximum body temperature of such an athlete is higher (32.5 °C) than that of an athlete who has not trained regularly (30.9 °C) and whose body probably requires further training.

## 1. Introduction

The survival of the system under changing environmental conditions depends on its adaptation abilities, i.e., on the entirety of functional reactions and structural changes aimed at maintaining broadly understood homeostasis. A good example of homeostatic and adaptive reactions that do not exceed the limits of proper regulation of the body’s functions is the body’s reaction to physical effort [1,2].

Endurance sports force, in some situations, make the greatest effort in a relatively short time, e.g., in the case of sprints. At the time, the necessary energy comes from anaerobic glycolysis. The consequence of this type of effort is therefore the accumulation of lactic acid in muscles and blood. The loads in anaerobic training are designed to adapt the athlete, which results in an increase in tolerance to acidification of the body and improvement of muscle fiber stimulation for extreme efforts [1]. The athlete’s return to physiological homeostasis after training is an important factor in body regeneration and has a significant impact on preventing overtraining [3,4,5,6]. The mechanisms of the organism’s return to thermal homeostasis seems to be associated with cardiovascular regeneration [1,2,7,8,9,10]. During exercise, the respiratory system must also adjust its work to the increased metabolic demand [11]. This entails increased consumption of oxygen, energy substrates, and increased production of carbon dioxide and heat. As a consequence, oxygen demand for muscles increases, which also increases blood flow. Physical effort triggers systemic adaptation processes, such as increase in cardiac minute volume and blood redistribution in the body [1]. Such processes are strongly connected with core temperature as well as body surface temperature [12].

The ventilation parameter VE increases already at the time of the first muscle contractions. The first, rapid growth phase is very short, followed by a transition phase that is milder. The next phase is the equilibrium phase, when the ventilation parameter stabilizes. The situation changes significantly in the case of progressive or high-intensity effort-ventilation increases and does not achieve clear stabilization. It is caused by the disturbance of the acid-base balance due to the increase of lactic acid concentration [9]. The volume of oxygen consumed by the body increases with the intensity of exercise and therefore with the increase of metabolism and oxygen demand of tissues. In performance tests, the VO_2_max parameter is determined based on the volume of oxygen taken in by the body, which is considered as an important indicator of the integrated function of the respiratory, cardiovascular, and muscular systems and lead to reflect in the changes in the body temperature.

When it comes to the cardiovascular response to moderate physical activity at a constant intensity, the so-called cardiovascular drift CVD is based on the progressive decrease in SV stroke volume and mean arterial pressure with a parallel increase in heart rate HR. During CVD, cardiac performance remains stable [13]. The cause of CVD is a decrease in the volume of water in the plasma, as a consequence of the body’s perspiration. This causes a decrease in venous blood volume and a decrease in cardiac output and ejection volume [1]. According to the American Heart Association, the resting heart rate (RHR) of a normal healthy person should be in the range of 60 to 100 bpm and its value decreases with age [14]. The situation changes when we are dealing with endurance athletes. The RHR value can be between 35 and 40 bpm, and this slowdown is called resting bradycardia. Bradycardia is accompanied by an increase in SV, which translates into a normal value of cardiac output. The simultaneous decrease in heart rate and increase in ejection volume are a beneficial symptom because they allow better filling of the ventricles with blood. Literature data also indicate that returning to RHR after exercise is more efficient in trained individuals [1,15].

Maximum heart rate HRmax can be reached by the body during maximum intensity exercise. However, recording your maximum heart rate must be preceded by a warm-up during progressive exercise. In addition, regeneration of the body plays an important role for this parameter, because reaching HRmax is possible for a rested body. Maximum heart rate is an individual trait, so HRmax values may be different for different athletes [1,11,15].

Thermal imaging is a non-invasive and non-contacting method to temperature measurements. However, a thermovision camera detects the heat radiation and changes it to the temperature distribution maps only from the objects’ surface. However, the inner body as well as environment conditions influence the surface temperature so by having the external conditions stabilized, it is possible to draw conclusions about the inner body processes (in the case of a living organism, we can draw conclusions about its changing metabolism, blood supply, etc.) [12]. According to the laws of physics, each body that has temperature higher than absolute zero (0K) radiates some energy in the infrared range [16]. Body homeostasis removes excess heat energy through the convection, conduction, radiation, and water evaporation mechanisms [2]. In fact, the body’s ability to heat radiation allows to use thermal imaging in many applications as some diagnostic tool. Thermal imaging is already used in many fields of medicine (e.g., oncology, dermatology, or stomatology). Nowadays, this method is becoming more and more popular in sports medicine, from injuries diagnosis to body temperature monitoring. It seems to be possible to use thermal imaging during and after the training in estimation some thermoregulation patterns from temperature parameters changes [1,7,16,17,18]. The analysis of an athlete’s fitness status requires the measurement of many parameters, which makes it quite complicated and tedious. Temperature measurement, on the other hand, allows you to easily view the condition of the athlete’s body. Therefore, in this pilot study we inquired on the use of thermovision measurements as instrumental to assess training status and adaptive changes in a sample of athletes.

## 2. Materials and Methods

### 2.1. Subjects

Two groups of athletes took part in the study. The main group was a group of cyclists subjected to effort before and after 1 training year. The reference group was a group of strength athletes subjected to one-time strength training using a rowing ergometer. The strength athletes due to specifics of theirs training and their stabilized high-level progression in the physical effort has been treated as a reference. Both groups contained 9 athletes. All subjects were health without any medical disorders. There were not reduces any samples.

Somatic features for groups are presented in Table 1. One can see that Strength group can be characterized by higher body mass parameter than cyclist group. Moreover, it can be noticed that average blood pressure for Cyclist group is lower than for a Strength group. These are typical differences due to the specific nature of analyzed sports.

### 2.2. Equipment

Training on rowing ergometer appears as a permanent point in the training program. Device like rowing ergometer provides general development training, involving the majority of muscle groups in the body (about 75–80%). The work carried out on the device is endurance-force, which boils down to an increase in overall fitness and condition. This makes strength athletes more adapted to training on an ergometer and, as a consequence, may suggest that the results obtained for this group can be treated as baseline. Performing work at a fixed pace and resistance classifies the effort on the "oarsman" for aerobic training [19].

There is a redistribution of blood flow in the body during exercise. This is caused by the expansion or narrowing of the arterioles that supply blood to specific organs. The highest increase in blood flow is noted in working skeletal muscles, due to an increase in tissue metabolism [5]. At rest, skeletal muscle blood flow is approximately 1200 mL/min. During very intense efforts, this value may increase to 12,500 mL/min and at maximum effort up to 22,000 mL/min [2].

To sum up, preparing an effective athlete’s training requires registering so-called "Physiological measures of effort." Therefore, athletes undergo endurance tests that assess the physical condition of the body. During the examination of the body’s efficiency, a number of parameters are determined simultaneously, ranging from easily measurable parameters, such as heart rate, and those whose measurement is more complicated, which determine the body’s abilities (e.g., lactic acid level, respiratory parameters) [1,2,20]. Thermographic analysis of the athlete’s body surface can become irreplaceable in the overall assessment of body performance, facilitating the interpretation of complicated parameters describing changes in body capacity and the selection of appropriate exercises.

Biomechanics of training on a rowing ergometer requires the use of force in a repetitive, maximum and smooth maneuver. The ergometer single maneuver can be divided into the following phases: the catch, the drive, leg emphasis, body swing emphasis, arm pull through emphasis, the finish, the recovery. Details about engaged muscle parts can be found in work (Mazzone, 1988) [19].

In addition, the choice of rowing ergometer as a test device is justified by the possibility of short-term efforts requiring the use of considerable force from volunteers. The training protocol selected in this way allowed to eliminate the sweat factor disturbing thermovision measurements. The research used the Concept 2 ergometer model D, which is one of the most popular rowing ergometers used by both professional athletes and amateurs, and at the same time, this model is extremely popular also in scientific works [21,22,23,24,25].

### 2.3. Procedures

The training protocol involved thermal imaging before, immediately after training, and 10, 20, 30, 40, and 50 min after training. By training is meant physical exercise on a rowing ergometer lasting 3 min and performed at the maximum efficiency of the athlete. Two groups of athletes performed the same protocol of exercises on a rowing ergometer in the same environmental conditions. Thermal imaging was performed using a Flir Systems T640 thermal imaging camera (FLIR Systems, Wilsonville, OR, USA) with a sensitivity of 0.03 K. The measurements were carried out in the Squashfit gym in Katowice-Ligota and CrossFit Dekerta in Krakow, where the climatic conditions in the training rooms were monitored. The test room temperature was 19.5 ± 0.7 °C and humidity 45.7 ± 3.6%. Each time, the volunteers were adapted to the ambient temperature for a period of 25 min. According to the guidelines of thermovision diagnostics in medicine, during the adaptation process, athletes did not change their body positioning, and the imaged body parts remained uncovered [26,27]. Volunteers were subjected to a test survey determining the use of painkillers or antipyretics in the last 24 h, taking antibiotics in the past week, alcohol consumption within 24 h before the test, taking sauna procedures within 2 days prior to the test and potential physical exertion on the day of the test. The research group consisted only of athletes who responded 100% negatively to the above questions.

For blood pressure measurement, a Marshall MB02 (OMRON Matsusaka Co. Ltd., Mie, Matsusaka, Japan) pressure gauge was used, while body weight and fat levels were measured using a TANITA UM-018 scale (Tanita Corporation, Tokyo, Japan) with 0.1 kg accuracy body and 0.1% for body fat. Thermal imaging was performed from a distance of 3.0 ± 0.1 m, and a thermal imaging camera was placed on a tripod.

### 2.4. Temperature Factors

**Figure 1:**
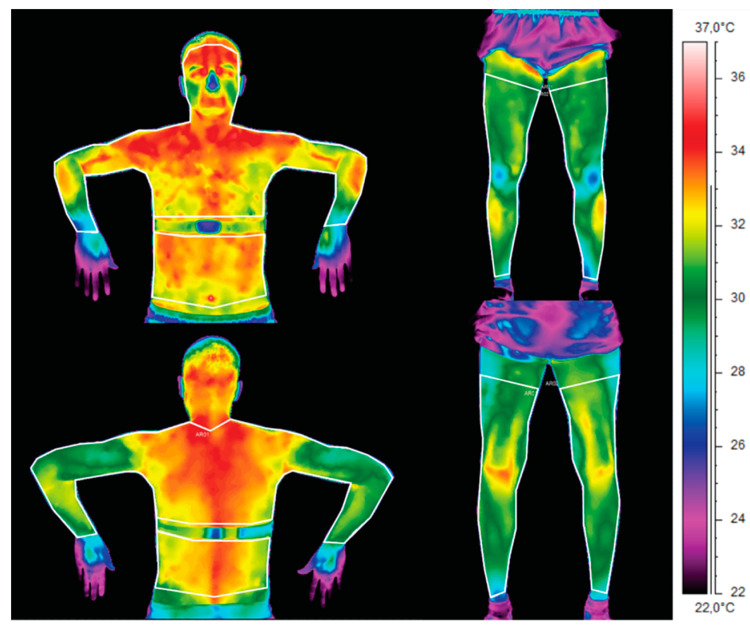
Areas of interest used to calculate the average body surface temperature for a representative athlete.

The average body surface temperature was calculated by averaging the ROI areas of interest shown in Figure 1, immediately before and after exercise on the rowing machine, as well as 10, 20, 30, 40 and 50 min after exercise. The surface temperature *T_pow_* was obtained by averaging the temperature value of all pixels (*i_T_*) from areas according to the Formula (1):

(1) $$T_{pow}=\sum i_{T}$$

As a consequence of the fact that body surface temperature strongly depends on psycho-physical conditions and environmental factors, for the purpose of deeper analysis, the temperature value was normalized to the value before exercise according to Formula (2) and marked as *T′_body_*:

(2) $$T_{body}^{\prime }=\frac{T_{i}}{T_{before}}$$

where *T_i_*–the body surface temperature at the time, *i* is before and after 10, …, 50 min.

The parameter *dT_max_* was calculated as the difference between the temperature before exercise *T_before_* and the maximum temperature after exercise *T_max post_* (3).

(3) $$dT_{max}=T_{before}-T_{max\;post}$$

The obtained thermal images were analyzed using the ThermaCAM TM Researcher Pro 2.8 SR-3 program and MS Office Excel 2016. The correlation analysis was performed with linear fitting. In the case of normal distribution and homogeneity of variances Student T-tests have been used. Opposite the Willcox’s tests were processed. Deeper analysis was done by using of Friedman’s ANOVA tests. For physiology and temperature parameter the correlation analyses were made at significance level *p* < 0.05 on PC program Statistica. All significance results were marked on graphs.

## 3. Results

**Figure 2:**
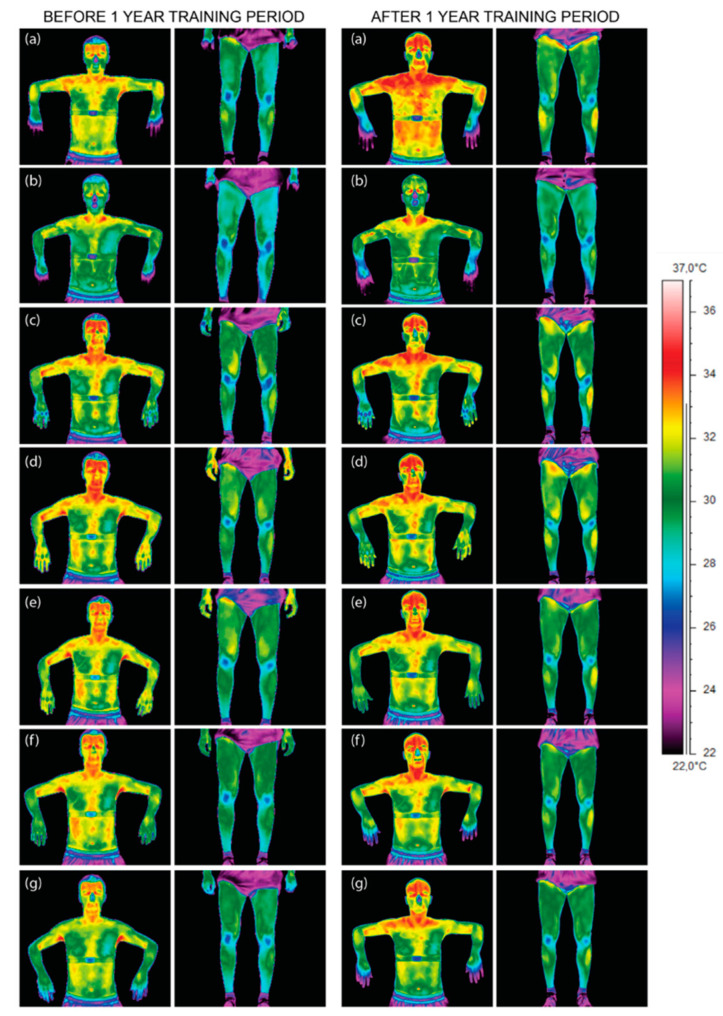
Thermal images of cycling volunteer’s chosen body parts before and after 1 year of professional training performed before (**a**), immediately after (**b**) and after 10 (**c**), 20 (**d**), 30 (**e**), 40 (**f**), and 50 min (**g**) of training.

The obtained thermal images of the front part of the body of an exemplary athlete indicate the occurrence of dynamic changes in the distribution of body temperature after exercise on a rowing ergometer. The observed changes in body surface temperature were associated with changes in skin blood flow and its modulation as a result of thermoregulation mechanisms. These changes could be estimated using the laws of heat exchange, which in turn could be used to assess the mechanisms of thermoregulation of competitors, depending on their training experience [1,7,28,29]. Figure 2 presents thermal images of a representative athlete, taking into account the phase of the training cycle (i.e., before and after 1 year of training) and the moment of imaging relative to training on a rowing ergometer with the assumed time intervals.

The initial visual assessment of the thermal images presented in Figure 2 did not indicate a clear relationship between the body temperature value and volunteer training experience. However, a clear difference in the values of the average output temperature of the athlete’s body can be observed by comparing a group of collages before the start of the annual training period and collages that have already completed this period. It seems that the observed differences in the body temperature of these two groups of athletes undertaking effort on an ergometer may be related to the other physiological state of their organisms obtained as a result of training. The values of physiological parameters are an indicator of the optimal efficiency of the body’s adaptation mechanisms, and therefore, it can be assumed that these groups differ in efficiency. The temperature depends on many factors, both external and internal, which may affect the measurement results [2,8,28]. The results obtained are presented as mean temperatures with standard deviation.

**Figure 3:**
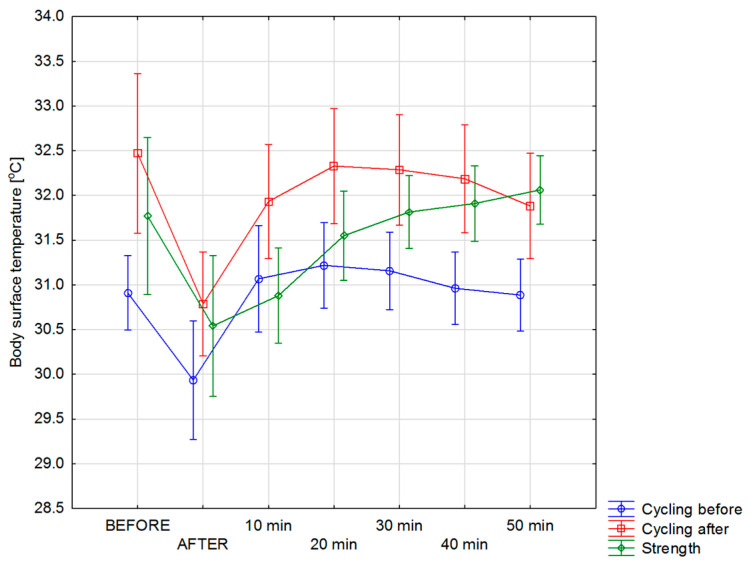
Mean body surface temperature changes obtained for studied group of cycling sportspersons before and after one training year and strength of sportspersons as a reference level performed before; immediately after; and after 10, 20, 30, 40, and 50 min of training.

The calculated average body surface temperature of individual groups of athletes is presented in Figure 3. For groups of cyclists before and after 1 year of training, the curves of changes in body surface temperature (red and blue curves) run similarly but have different amplitudes at individual time intervals of the conducted tests. However, for the strength group, the temperature dependence seems to be a little different than cycling. Comparing it to the groups of athletes analyzed before and after the annual training, it is clear that in the strength group, there are differences in the course of the temperature curve, as well as in the amplitude of these changes.

In both groups of cyclists (red and blue line) the body surface temperature tends to increase up to 20 min after exercise, followed by some temperature stabilization process. In the case of the strength group, the upward trend in body temperature is observed up to 50 min after exercise. Analysis of the data presented in Figure 3 indicates, however, that there is a big difference, as much as 1.56 °C at the initial temperature, for groups of cyclists before and after a year of training, before training on an ergometer.

**Figure 4:**
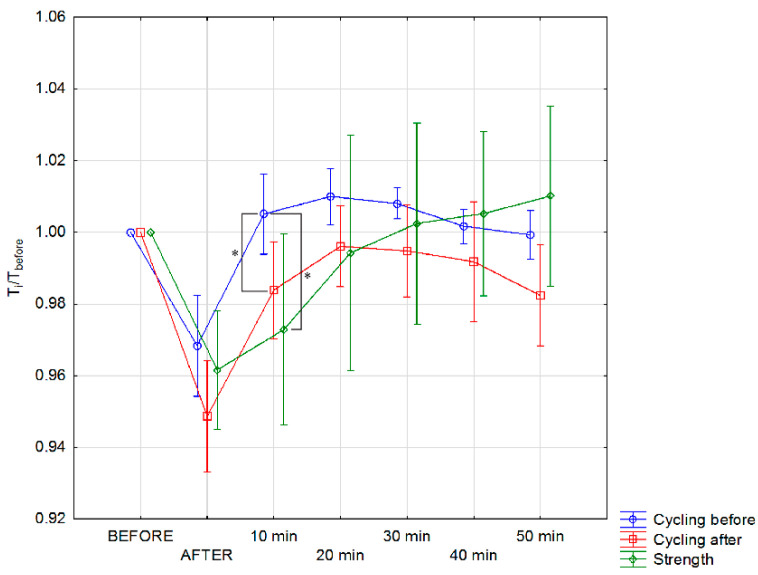
Normalized mean body surface temperature changes for studied group of cycling sportspersons before and after one training year and strength of sportspersons as a reference level performed before; immediately after; and after 10, 20, 30, 40, and 50 min of training. Statistically significant changes were marked as * on the graph with *p* < 0.05.

The normalized body surface temperature *T′_body_* defined in Equation (2) for individual groups, before making an effort on an ergometer and at particular time intervals of this training, is presented in Figure 4.

Despite the small differences obtained between the shape of the curves for *T_body_* (Figure 3) and *T’_body_* (Figure 4), it is worth noting that the normalized temperature graph better reflects changes in body surface temperature values in individual training groups. It is clearly seen that the body surface temperature after exercise on an ergometer for a group of cyclists before the training season, takes higher values relative to the initial temperature, up to 40 min after exercise. After 1 year of training, the temperature after exercise does not exceed the initial value.

In turn, the body surface temperature of the strength group, takes higher values than the initial temperature, starting from 30 min after exercise and during subsequent measurements (i.e., 40 and 50 min after exercise) continues to increase. It should be also seen that there are significant differences between normalized mean body surface temperature obtained for cyclist before and strength as well as cyclist before and cyclist after observed in 10 min after training. Such observation seem to may bring indirectly some information about sportspersons efficiency due to differences of body temperature rise after 10 min when sweat does not play main role in the surface temperature. This phenomenon is correlated with metabolism level so the body thermoregulation efficiency.

**Figure 5:**
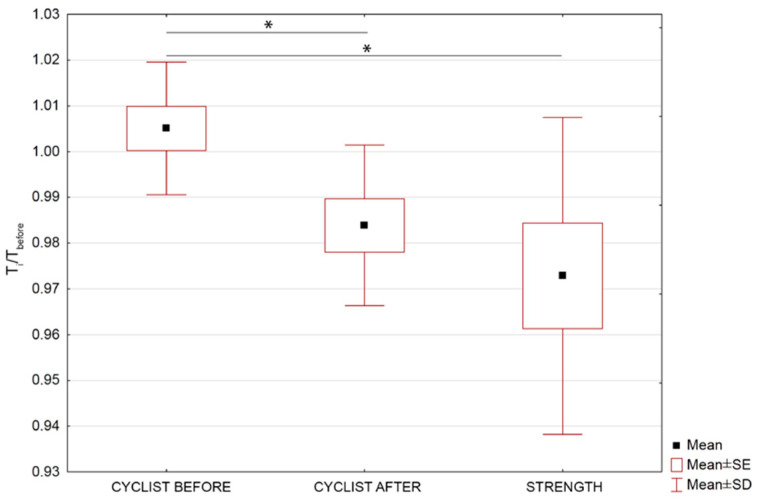
Normalized mean body surface temperature changes for studied group of cycling sportspersons before and after one training year and strength of sportspersons as a reference level performed after 10 min of training. Statistically significant changes were marked as * on the graph with *p* < 0.05.

**Figure 6:**
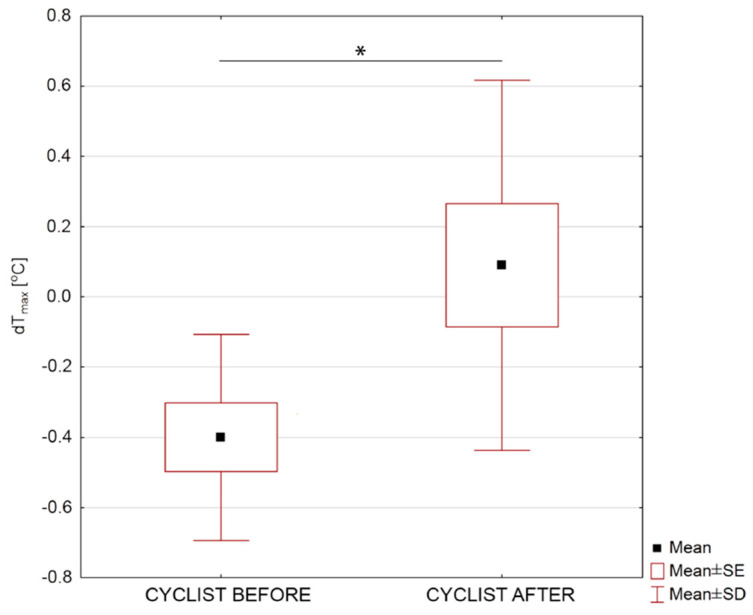
Maximum body surface temperature difference for studied group of cycling sportspersons before and after one training year. Statistically significant changes were marked as * on the graph with *p* < 0.05.

Analyzing Figure 3 and Figure 4 it can be seen that for groups of cyclists before and after 1 year of training, there were changes in the pattern so the temperature values too in the body surface temperature after exercise. That is why the deeper analysis have been done and its results are shown in Figure 5 and Figure 6 and discussed later.

The values of the *dT_max_* parameter defined in Equation (3) for individual groups of cyclists are presented in Figure 6 taking into account statistically significant differences for *p* < 0.05.

According to the data presented in Figure 6, the value of the *dT_max_* parameter for a group of cyclists before 1 year of training was −0.4 °C, while after 1 year of training, 0.1 °C. Therefore, after 1 year of training, the maximum temperature after exercise on the ergometer assumes a lower value than the initial temperature, i.e., before exercise. However, before the training season, the temperature value after exercise takes a higher value than the initial value. In addition, the parameter difference between the groups was verified using the Student’s T test, and the obtained probability value *p* = 0.019 was statistically significant difference.

This may indirectly indicate a change in the thermoregulation pattern of the body, as a result of adaptation processes in the body by implementing endurance training within 1 year.

**Figure 7:**
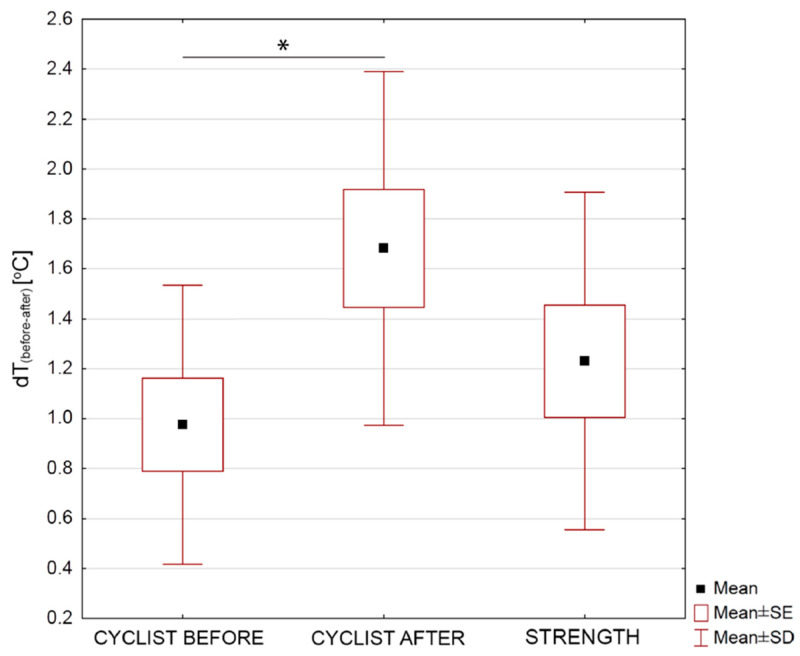
Body surface temperature coefficient *dT_(before-after)_* (defined as body temperature before-body temperature after training) difference for all studied groups: cycling sportspersons before and after one training year as well as strength sportspersons group. Statistically significant changes were marked as * on the graph with *p* < 0.05.

For further analysis of the differences between the groups of cyclists, the parameter *dT_(before-after)_* was calculated, determining the temperature difference before and after the exercise test on the ergometer, in order to check how the body surface temperature changed immediately after training, relative to the reference temperature, i.e., before training. The results are shown in Figure 7.

The data presented in Figure 7 clearly show that for a group of cyclists after 1 training year, the difference in the value of the *dT* parameter (before-after) is statistically significant with *p* = 0.007. The decrease in body surface temperature as a result of dynamic physical exertion is greater after a year of training. The difference for the group of cyclists before and after the training season is 0.7 °C with a probability factor *p* < 0.05. There were no statistically significant differences between the groups of cyclists and the strength group. Based on Figure 7, it can be assumed that for cyclists after the training season, the lowering of body temperature immediately after training is greater than in the group of cyclists before starting the training year, which may suggest an increase in the effectiveness of thermoregulation mechanisms [29,30].

**Figure 8:**
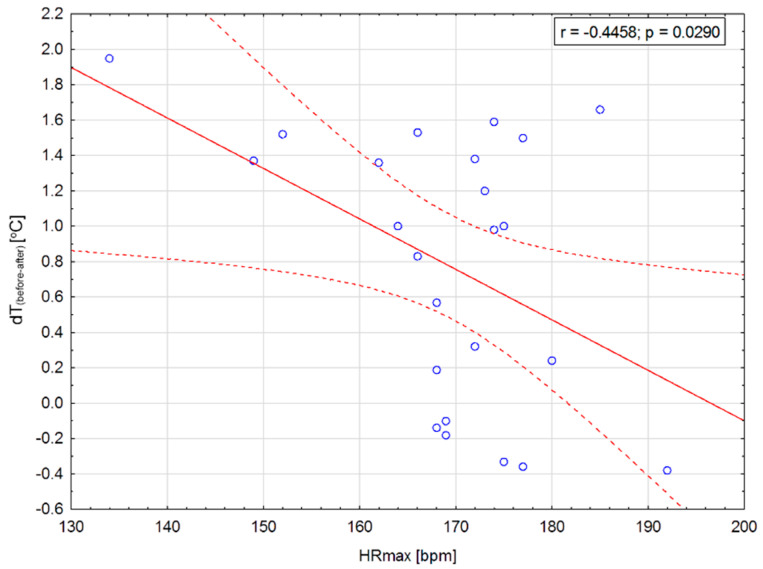
Correlation between the body surface temperature coefficient *dT* (defined as body temperature before-body temperature after training) and maximum heart rate obtained for all measured subjects.

The above assumptions can be confirmed by the data presented in Figure 7, namely the correlation between the maximum heart rate HR_max_ and the parameter *dT_(before-after)_*. The results are shown in Figure 8.

Based on the data presented in Figure 8, it can be seen that between the maximum heart rate HR_max_ and the parameter of the difference in body surface temperature *dT_(before-after)_*, there is an average negative correlation with a Pearson’s coefficient equal to −0.45. This relationship is statistically significant, and the significance factor is *p* = 0.03. The relationship between HR_max_ and *dT_(before-after)_* is negative, which indicates that the higher the maximum heart rate during training, the lower the temperature drop after exercise. The high maximum heart rate is characteristic for young and untrained athletes [1]. Hence, it can be concluded that the value of *dT_(before-after)_* corresponds directly with the effectiveness of thermoregulation mechanisms. It seems that low *dT_(before-after)_* values will be typical for less-trained athletes, and as this parameter increases, the body’s fitness will increase.

The obtained Pearson’s coefficient *r* = −0.45 shows that correlation is negative and average with p factor less than 0.05. That indicate some relationship may occur between one of the most important physiological parameter (HR_max_) and the temperature coefficient.

## 4. Discussion

Based on changes in body surface temperature, it can be assumed that since endurance training affects the development of athlete’s physiological adaptation processes, it will also affect the efficiency of thermoregulation mechanisms. Moreover, as a result of training, the LT (lactate threshold) increases, which allows you to do exercises of higher intensity without significant signs of fatigue. The LT lactate threshold is defined as the intensity of exercise (e.g., running speed) above which the lactate concentration in the blood rises above the resting level.

The increase in lactate concentration occurs when its production exceeds the capabilities of the mechanisms responsible for its removal. In addition, a modification in muscle blood flow and an increase in tissue oxygen efficiency are occurring [1,9,31]. Modulation of blood flow, better oxygenation of tissues and changes in the efficiency parameters of the body can also significantly affect thermal comfort and the effectiveness of the body’s thermoregulation mechanisms.

The conducted studies of changes in body surface temperature after a short and dynamic training show clear differences in the course of curves between groups of cyclists and a strength group. The observed differences in the course of temperature changes between the groups of cyclists and the strength group seem to be connected with different the specificity of training in individual groups. The period of 1 year of training between measuring groups of cyclists showed a slightly different pattern of temperature stabilization after exercise. This can be caused by the development of the body’s adaptive mechanisms and the occurrence of changes in skin and muscular blood flow, which have been described in the literature. It is known that endurance training induces many physiological adaptations. In the case of the cardiovascular system, there is a reduced heart rate and an increase in the ejection volume of the heart. This workout increases the LT threshold, which allows you to do higher intensity exercises without a significant increase in lactic acid levels. It causes a lowering of the resting heart rate RHR. A well-planned training allows VO_2_max to increase by 10 to 15% for beginners. The blood flow through the muscles is modified, which results in a better use of oxygen by the muscle cells, as the number of capillaries in the tissue increases. In addition, there is an increase in mitochondrial density and oxidative enzyme activity [1,9,31].

The analyzed results of the body surface temperature assessment and the thermal parameters proposed in the analysis seem to be in accordance with literature reports. The relationships between the parameters of temperature differences in groups of cyclists assume statistically significant values from *p* < 0.05, which may indirectly confirm the changes that occurred in the body thermoregulation mechanisms. In addition, the occurrence of an average negative correlation of *p* < 0.05 between the parameter of the temperature difference before and after exercise and maximum heart rate confirms the usefulness of thermovision, in an indirect assessment of the effectiveness of the mechanisms of return to thermal homeostasis of athletes, depending on the degree of their training. Based on the results obtained, it can be assumed that as the body’s efficiency increases, the effectiveness of thermoregulation mechanisms also increases.

Nowadays there are many articles about the applications of thermal imaging in sports medicine. It seems that thermal imaging as a method of temperature measurement can give some benefits like general overview of temperature pattern changes due to training, use IRT to diagnose sports injuries, monitoring the muscles regeneration after training or assessing the effects of different environment conditions [7,32,33,34]. However, the analyzing temperature parameters in terms of sports efficiency evaluation seems to be quite interesting and innovative. As it was mentioned before training induces many adaptations processes, which may become some indicators of athlete’s efficiency and protect him from overtraining or some serious injuries. In fact, it is necessary to find some easy and non-invasive method for efficiency monitoring during the training plan realization. It seems that thermal imaging may become a technique helpful for training monitoring by using some temperature parameters as indicators of efficiency level and overtraining predictor [8,30].

The data presented are, however, preliminary reports, and further analysis is necessary, taking into account the measurement of significant parameters, such as VO_2_max and lactic acid level and their subsequent correlation with temperature parameters.

## 5. Conclusions

Performed studies showed significant differences between values of normalized mean body surface temperature obtained for cyclists before and after the training period as well as cyclists before the training period and strength observed 10 min after training. Such observation may bring indirectly some information about the sportspersons’ efficiency due to differences in body temperature change after 10 min training when sweat does not play a main role in surface temperature. Observed mean body temperature drop due to training was nearly 1 °C. It concludes that thermovision not only allows to monitor body temperature changes due to activity but also to determine which of the athletes has a high level of body efficiency. The average maximum body temperature of such an athlete is higher than that of an athlete who has not trained regularly, and probably his or her body requires further training.

## Figures and Tables

**Table 1:** Somatic features of athletes divided into groups.

Group	Period of Study	Number of Samples	Average Age [years]	Seniority [years]	Average Body Weight [kg]	BMI [kg/m^2^]	Average Body Fat Content [%]	Average Blood Pressure [mmHg]
Cyclists	Before Training Period	9	27 ± 3	6 ± 2	72.1 ± 5.2	22.6 ± 1.6	14.7 ± 3.6	124/76
	After Training Period	9	28 ± 3	7 ± 2	73.1 ± 6.0	22.9 ± 1.4	15.6 ± 3.4	127/76
Strength	Reference	9	25 ± 4	8 ± 3	83.0 ± 10.8	26.0 ± 3.1	18.6 ± 3.0	138/81
